# Sirolimus therapy following subtotal pancreatectomy in neonatal hyperinsulinemic hypoglycaemia: a case report

**DOI:** 10.1186/1687-9856-2015-S1-P81

**Published:** 2015-04-28

**Authors:** Mary Abraham, Sarah Flanagan, Vinutha Shetty, Glynis Price, Martin deBock, Aris Siafarikas, Sian Ellard, Steven Resnick, Elizabeth Whan, Elizabeth Davis, Timothy Jones, Khalid Hussain, Catherine Choong

**Affiliations:** 1 Department of Endocrinology, Princess Margaret Hospital, Perth, WA, Australia; 2 Institute of Biomedical and Clinical Science, University of Exeter Medical School, UK; 3 School of Paediatrics and Child Health, UWA, Perth, WA, Australia; 4 Department of Neonatology, Princess Margaret Hospital, Perth, WA, Australia; 5 Department of Surgery, Princess Margaret Hospital, Perth, WA, Australia; 6 Telethon Kids Institute, UWA, Perth, WA, Australia; 7 Clinical and Molecular Genetics Unit, Great Ormond Street Hospital for Children, London, UK

## 

Hyperinsulinemic hypoglycaemia (HH) occurs due to an unregulated insulin production from the pancreatic β-cells in the presence of low blood glucose. Mutations in ABCC8 and KCNJ11 are associated with severe HH that is unresponsive to conventional medical treatment. The only treatment for patients with medically unresponsive diffuse HH is a subtotal pancreatectomy. However, following surgery, hypoglycaemia may persist and some patients develop diabetes and malabsorption. Overexpression of the mTOR pathway is contributory to HH. Sirolimus, an mTOR inhibitor, is currently used in the treatment of congenital hemangiomas and in post renal transplant. Senniappan et al [1] recently reported efficacy of sirolimus in four surgically naïve patients with diffuse HH unresponsive to diazoxide and octreotide.

We present a patient who was treated with sirolimus due to persistent hypoglycaemia following subtotal pancreatectomy.

A term neonate with a birth weight of 4.67kg had persistent hypoglycaemia since birth secondary to hyperinsulinism and was unresponsive to treatment with maximal doses of diazoxide and octreotide with a glucose infusion requirement (GIR) of 38mg/kg/min. Genetic testing revealed a homozygous ABCC8 nonsense mutation, p.Gln1020Ter. A subtotal pancreatectomy was performed on day 40. Post-surgery, he had a GIR of 20mg/kg/min and was recommenced on daily subcutaneous (SC) octreotide, with monthly long acting (LA) octreotide. At 3 months, he was commenced on oral sirolimus. The dose was adjusted to maintain serum trough levels between 5 and 15ng/ml. Parenteral fluids and SC octreotide were weaned over a month. He was discharged home at 4.5 months on sirolimus (2.5mg/m^2^/day) and LA octreotide. He was monitored with capillary blood glucose testing twice a day with the aim to maintain levels above 3.5mmol/L. Further surgery has been deferred. His growth and development are appropriate at 6 months of age with no side effects from sirolimus.

The clinical response in our patient supports sirolimus as a new therapeutic strategy in patients with HH which may facilitate deferment of surgery.

Written informed Consent for this patient has been taken including results of the genetic analyses and histopathological images according to the Institutional Ethics Committee procedures of our health service
